# Long-term robot-assisted gait training in patients with cerebral palsy: meaningful changes in gait parameters require time

**DOI:** 10.3389/fnhum.2026.1924639

**Published:** 2026-09-24

**Authors:** Paweł Chmara, Sabina Brazevič, Marek Jóźwiak, Faustyna Manikowska

**Affiliations:** Gait and Motion Analysis Laboratory, Department of Pediatric Orthopedics and Traumatology, Poznań University of Medical Sciences, Poznań, Poland

**Keywords:** cerebral palsy, gait endurance, gait parameters, Gross Motor Function Classification System, robotic-assisted gait training

## Abstract

**Introduction:**

Robot-assisted gait training (RAGT) is increasingly used in children with cerebral palsy (CP), but evidence regarding the timing and persistence of meaningful changes in gait remains limited. This study aimed to describe long-term changes in spatiotemporal gait parameters, gait kinematics, and walking endurance in ambulatory patients with CP undergoing systematic, repetitive RAGT, and to determine whether the timing of clinically meaningful change differs between ambulatory levels.

**Methods:**

A study group consisting of 211 patients with hypertonic CP (93 females, 118 males; mean age 10.33 ± 3.80 years) classified at Gross Motor Function Classification System (GMFCS) levels I–III underwent a structured 2-year RAGT program comprising four therapy sets and 40 sessions in total. Gait was assessed five times: at baseline (pre-intervention) and at 6-month intervals over 24 months. Outcomes included the Gait Deviation Index (GDI), spatiotemporal gait parameters obtained from three-dimensional gait analysis, and six-minute walk test (6MWT) distance. The follow-u*p* values were compared with the baseline. Clinical meaningfulness was evaluated using the minimal clinically important difference (MCID) thresholds.

**Results:**

In the entire group, step length and stride length increased significantly from T2 onward, walking speed from T3 onward, and 6MWT distance at each follow-up assessment. At T5, individual improvement exceeding the relevant MCID thresholds was observed in 61.61% of participants for step length, 62.09% for stride length, 52.13% for walking speed, and 51.66% for 6MWT distance. Cadence increased significantly only at T5, whereas GDI increased throughout follow-up; however, the corresponding MCID thresholds were exceeded by 32.23 and 46.92% of participants, respectively. GMFCS III showed the earliest and largest gains, while changes in GMFCS II generally emerged later. Effect sizes increased over time and reached their highest values at T5. Repeated-measures ANOVA confirmed significant time effects for most outcomes. Significant time-by-GMFCS interactions for cadence, step length, and 6MWT distance indicated that selected longitudinal trajectories differed across functional levels, although the interaction effect sizes were small.

**Conclusion:**

Systematic, repetitive gait training based on RAGT in patients with CP was associated with long-term positive changes in spatiotemporal gait parameters and walking endurance. In contrast, clinically meaningful changes in gait kinematics, as quantified by the GDI, were limited across all GMFCS levels. The effects of systematic and repeated RAGT developed progressively over time, with selected outcomes following different trajectories according to the participants’ functional level.

## 1 Introduction

Cerebral palsy (CP) is a group of disorders affecting the development of movement and posture, leading to limitations in activity, attributed to non-progressive disturbances occurring in the developing fetal or infant brain. These motor disorders are often accompanied by disturbances of sensation, cognition, communication, perception, and/or behavior, and/or seizure disorder (Bax et al., 2005).

The nature of these disorders is permanent, with symptom onset occurring in the early development and resulting in lifelong consequences, and there is currently no definitive cure (Graham et al., 2016). CP is a non-progressive neurological disorder, however it can progress without proper treatment severity of motor impairments leading to reduced physical activity and future complications in adulthood (Morgan and McGinley, 2014; Verschuren et al., 2016).

Based on the International Classification of Functioning, Disability and Health (ICF) framework (World Health Organization, 2001), a shift in treatment concepts has occurred over the past decade – from interventions targeting a single-impairment (such as restricted range of motion, weakness or abnormal muscle tone) and anticipating non-participation-based interventions, toward treatment aimed at improving body function, activity, and participation as a main goal (Novak et al., 2020). It has been shown that only 24% of interventions for CP are proven to be effective, while the effects of 70% are uncertain and remaining 6% are ineffective. Moreover, effective interventions typically affect only a single ICF level (Novak et al., 2013).

To date, scientific literature supports the effectiveness of functional therapy training, characterized by the execution of task-specific movements associated with activities of daily living, as the most appropriate approach for promoting gross motor function in CP (Christy et al., 2012). This approach aligns with a better understanding of motor-learning processes, which indicate that repetitive, task-specific movements lead to the reorganization of motor pathways and accelerate skill development (Barbeau, 2003; Hubbard et al., 2009).

Through repetitive task-specific practice and the promotion of physiological-like movement patterns robot-assisted gait training (RAGT) enhances neuroplasticity and drives skill acquisition (Winchester et al., 2005; Luft et al., 2008). RAGT enables therapy tailored to the patient’s specific needs by individually adjusting levels of difficulty, degrees of body-weight support, and guidance force. RAGT is easy to monitor, goal-directed gait training that is also cognitively engaging (Swinnen et al., 2010; Beretta et al., 2020). A wide variety of RAGT devices is used in both clinical practice and research. These include systems combining orthoses with the adjustable body-weight-supported harness, which allows assisted treadmill walking, as well as different models of exoskeletons, wearable robots, hybrid assistive limbs, gait trainers or ergometers with motorized footplates (Swinnen et al., 2010; Beretta et al., 2020; Bunge et al., 2021; Cortés-Pérez et al., 2022; Chen et al., 2026).

An expanding body of scientific literature provides evidence supporting the effectiveness of RAGT in children (Bunge et al., 2021; Cortés-Pérez et al., 2022; Chen et al., 2026). However, previous studies were inconsistent with respect to patient characteristics, and training dosage has varied widely due to differences in the equipment used. Consequently, conclusions regarding the impact of RAGT on the functional status of children with CP remains inconclusive.

The overall intent of using RAGT is to modulate the level of participation and degree of body-weight support required during functional gait according to their therapeutic goals (Swinnen et al., 2010), which can be achieved by using different RAGT devices. However, it remains unclear what longitudinal changes in gait can be observed in patients with CP, how long these changes persist, and how long does it take for the changes to appear.

Therefore, the aim of the present study was to describe the changes in gait spatiotemporal parameters and gait endurance in patients with CP undergoing structured, repetitive gait training based on RAGT. In addition, the study aimed to evaluate the dynamics of changes over time, and to determine if ambulatory level affects responsiveness to RAGT.

## 2 Materials and methods

### 2.1 Participants

211 patients with hypertonic CP (female: 93; male: 118, age: 10.33 ± 3.80 years) were recruited for the study. Patients were able to walk independently or with an assistive device and were classified according to the Gross Motor Function Classification System (GMFCS) as level I (n = 55), level II (n = 113), and level III (n = 43).

To be enrolled in the study, participants had to meet the inclusion criteria: 1. diagnosis of CP; 2. no surgical interventions within 1 year; 3. no botulinum toxin injections within past 6 months; 4. ability to follow verbal instructions; and 5. ability to walk without physical assistance. Exclusion criteria included the following: 1. pain; 2. leg length discrepancy and/or fixed contractures that precluded RAGT; and 3. time interval greater than 6 months between RAGT sessions.

Participants were enrolled in the study while already receiving conventional therapy. This therapy was neither discontinued nor modified because of study participation and was continued at the same level throughout the follow-up period.

Data were collected at a RAGT outpatient clinic of a local rehabilitation hospital. All assessments were performed in the Motion Analysis Laboratory. The study protocol was approved by the Institutional Review Board, and written informed consent was obtained from all participants aged 18 years or older, as well as from the parents or legal guardians of underage participants.

### 2.2 Protocol

Each participant completed 40 RAGT sessions in total over a two-year period. The therapy was administered in two-week blocks and repeated every six months. Each daily program lasted 180 min, including approximately 40 min of scheduled rest between therapeutic activities, and consisted of:

1 a warm-up phase with strengthening exercises;
2 three 20-min sessions of balance training on specialized platforms;
3 two 30-min RAGT sessions individualized to the patient’s abilities and therapeutic goals;
4 a cool-down phase with stretching.

The equipment used for RAGT was tailored to each patient’s physical requirements, body size, and functional capacity. Equipment selection was reassessed before each new set of therapy sessions, but no more often than every six months. Physical therapists (PT) with at least one year of experience in RAGT selected the appropriate equipment from the following options:

- Powered exoskeleton EksoGT (Ekso Bionics Holdings, Inc., San Rafael, CA, United States). The device is designed for users with a body weight of up to 100 kg, a height range of 1.58–1.88 m, and a maximum hip width of 45.7 cm. The system weighs 27 kg and includes two lithium-ion batteries (2 Ah, 48.1 VDC) capable of producing a peak current of 30 A, allowing up to 60 min of full-load operation. The EksoGT supports active hip flexion from −20° to 135° and passive hip abduction from −2° to 4°. Knee flexion is motor-assisted within a range of 0°–135°, while the ankle allows passive plantarflexion from −10° to 10°. Foot stiffness can be adjusted on a scale from 1 (flexible) to 4 (rigid). The device enables a walking speed of approximately 2 km/h, with adjustable step lengths (20.3–45.7 cm), step widths (0.0–7.6 cm), and swing phase duration (0.8–2.5 s).
- Driven gait orthosis Lokomat Pro Pediatric (Hocoma, Volketswil, Switzerland). The system provides treadmill-based, RAGT with powered hip and knee joints and dynamic body-weight support. The pediatric orthosis fits children with femur lengths of 21–35 cm and pelvic width 17–28 cm, while the standard (adult) orthosis covers femur length of 35–47 cm and pelvic widths of 29–51 cm. The treadmill operates at speeds ranging from 0 to 10 km/h, with Lokomat-assisted training speeds up to 3.2 km/h. Body-weight support is electrically controlled with static/dynamic unloading and continuous adjustment up to 85 kg; the maximum patient weight is 135 kg.
- End-effector gait trainer G-EO (Reha Technology, Olten, Switzerland). The gait re-education device with distal fixation of the feet to two programmable footplates and suspension in a body-weight-supported harness. It is designed for level-ground walking training as well as for simulating stair ascent and descent. Operating modes include Active mode, in which the patient initiates movement after overcoming a preset resistance threshold, and Active-Assistive mode, in which movement initiation is detected and facilitated by the device. Level-walking trajectories and stair-climbing patterns are based on established scientific data. Actuation is located within the footplates and allows a maximum walking speed of 2.3 km/h, a cadence of up to 70 steps/min, a step length of up to 55 cm, a stair step height of up to 20 cm, and a foot angle adjustment range of −10° to +10°. The device is intended for patients with heights of 140–200 cm when using standard footplates or 90–160 cm with pediatric footplates, and a maximum body weight of 150 kg.

Device selection was based initially on compatibility with the technical specifications of each system, including the participant’s height, body weight, pelvic or hip width, and femur length. Among technically compatible devices, physical therapists selected the equipment according to the participant’s functional abilities, required level of body-weight support and movement assistance, therapeutic goals, and safety considerations. The selected combination of devices remained unchanged throughout each two-week RAGT set but could be reassessed before the subsequent set if the participant’s body dimensions, functional abilities, or therapeutic needs had changed.

Gait assessment was performed five times at six-month intervals:

- T1 (baseline): within 1 week before the first set of RAGT sessions;
- T2: 6 months after baseline assessment when the first RAGT cycle was completed and within 1 week before the second set of RAGT sessions;
- T3: 12 months after baseline assessment, following completion of the second RAGT cycle and within 1 week before the third set of RAGT sessions;
- T4: 18 months after baseline assessment, following completion of the third RAGT cycle and within 1 week before the fourth set of RAGT sessions;
- T5: 24 months after baseline assessment, corresponding to approximately 6 months after the fourth RAGT cycle.

Each evaluation included gait quality (GDI), and spatiotemporal parameters obtained using instrumented three-dimensional gait analysis and the six-minute walk test (6MWT). Each follow-up result was evaluated in relation to the baseline (T1), rather than between consecutive visits. Therefore, comparisons between T2 and T1, T3 and T1, T4 and T1, and T5 and T1 were made.

Kinematic data were collected using an infrared 8-camera three-dimensional gait analysis system (six Bonita 3 cameras and two Vero 2.2 cameras; Vicon Motion Systems Ltd., UK) sampling at 120 Hz. Reflective markers were placed on each patient’s body according to the Lower Body Plug-in-Gait model protocol (Davis et al., 1991). Participants walked barefoot along a 10 m walkway at a comfortable, self-paced speed.

During the 6MWT, participants were instructed to walk at their maximum sustainable speed for six minutes along a 15 m course marked on a flat, cone-delineated surface, with each meter of the path labeled. The examiner recorded the duration using a stopwatch and the total distance was calculated as the sum of the completed laps and any additional full meters covered in the final, incomplete lap (Enright, 2003).

### 2.3 Data analysis

#### 2.3.1 Outcome measures

Primary outcome measures included the following spatiotemporal gait parameters: cadence [step/min], walking speed [m/s], stride length [m], step length [m], step width [m], as well as 6MWT distance [m] and GDI. The clinical meaningfulness of change was evaluated using the minimal clinically important difference (MCID) framework (Jaeschke et al., 1989; Revicki et al., 2006).

For spatiotemporal gait parameters and 6MWT distance, the MCID was estimated for each group using a distribution-based threshold of 0.5 standard deviation (MCID = 0.5 × SD) (Norman et al., 2003). In groups with non-normal distributions, SD was approximated from the interquartile range using an IQR-based estimator (σ_IQR_ = IQR/1.349) (Hoaglin et al., 2000).

For the GDI, an MCID of 5 points was adopted as reported in the literature (Schwartz et al., 2016).

Because distribution-based MCID estimates have recognized limitations, a supplementary responder analysis was performed. Participants were classified as showing improvement (Δ ≥ MCID), no clinically meaningful change (|Δ| < MCID), or deterioration (Δ ≤ −MCID), where Δ represents the change in the analyzed parameters compared to baseline (T1) (Draak et al., 2019; Malec and Ketchum, 2020; Dekker et al., 2024).

#### 2.3.2 Statistical analysis

Within-subject changes between study visits were assessed using paired statistical tests. Paired t-tests were applied to variables with a normal distribution and homogeneity of variance, whereas the Wilcoxon signed-rank test was used when these assumptions were not met. To account for multiple comparisons, *p* values were adjusted using the Benjamini–Hochberg procedure to control the false discovery rate.

In addition, the magnitude of change from baseline was quantified using Hedges’ *g_z_*, with 95% confidence intervals estimated using bootstrap resampling. Pairwise comparisons of each follow-up assessment with baseline were adjusted using the Holm procedure. The longitudinal effect of time was evaluated using repeated-measures analysis of variance (ANOVA) for the entire study group and separately for each GMFCS subgroup. Differences in longitudinal trajectories across GMFCS levels were assessed using the time-by-GMFCS interaction. Eta squared (η^2^) was reported as the measure of effect size for the main effect of time and the time-by-GMFCS interaction.

All analyses were performed using Statistica version 14 (TIBCO Software Inc., USA). Statistical significance was set at *p <* 0.05.

## 3 Results

In the entire study group, step length and stride length increased significantly during follow-up, from 0.42 m to 0.48 m and from 0.84 m to 0.96 m, respectively (Table 1, Figure 1). Walking speed increased significantly from T3 onward and reached 0.90 m/s at T5, while 6MWT distance increased significantly at each follow-up assessment. At T5, individual improvement exceeding the relevant MCID thresholds was observed in 61.61% of participants for step length, 62.09% for stride length, 52.13% for walking speed, and 51.66% for 6MWT distance. Cadence increased significantly only at T5, with 32.23% of participants exceeding the MCID threshold. GDI also increased significantly, but the proportion of participants exceeding the adopted MCID threshold reached a maximum of 46.92% at T5. Step width remained unchanged throughout the study period.

**Table 1:** Spatiotemporal parameters in the entire study group.

Parameter	MCID	T1	T2	T3	T4	T5	T2 to T1	T3 to T1	T4 to T1	T5 to T1
		Mean (SD) or median (IQR)	Mean (SD) or median (IQR)	Mean (SD) or median (IQR)	Mean (SD) or median (IQR)	Mean (SD) or median (IQR)	*p* value (% ≥ MCID)	*p* value (% ≥ MCID)	*p* value (% ≥ MCID)	*p* value (% ≥ MCID)
Cadence [step/min]	10.29	107.00 (28.25)	105.00 (26.00)	107.50 (26.75)	108.00 (21.85)	110.00 (24.70)	0.773 (21.33)	0.728 (25.12)	0.501 (28.91)	**0.018** (32.23)
Walking speed [m/s]	0.14	0.76 (0.38)	0.72 (0.26)*	0.81 (0.39)	0.86 (0.38)	0.90 (0.39)	0.282 (26.07)	**< 0.001** (29.86)	**< 0.001** (42.65)	**< 0.001** (52.13)
Stride length [m]	0.10	0.84 (0.27)	0.83 (0.21)*	0.89 (0.25)	0.91 (0.22)*	0.96 (0.28)	**0.019** (26.54)	**< 0.001** (39.81)	**< 0.001** (51.18)	**< 0.001** (62.09)
Step length [m]	0.05	0.42 (0.13)	0.42 (0.11)*	0.45 (0.12)	0.46 (0.11)*	0.48 (0.14)	**0.011** (29.86)	**< 0.001** (40.76)	**< 0.001** (53.08)	**< 0.001** (61.61)
Step width [m]	0.03	0.14 (0.08)	0.15 (0.07)	0.15 (0.08)	0.14 (0.08)	0.15 (0.07)	0.140 (17.06)	0.111 (19.43)	0.770 (22.75)	0.888 (19.43)
GDI [−]	5.00	79.84 (16.39)	80.67 (17.28)	81.46 (16.75)	82.39 (15.76)	82.91 (16.69)	**0.001** (30.80)	**< 0.001** (33.65)	**< 0.001** (35.07)	**< 0.001** (46.92)
6MWT distance [m]	51.70	363.00 (139.50)	373.00 (139.00)	378.00 (128.00)	376.00 (142.00)	390.88 (128.33)*	**< 0.001** (30.33)	**< 0.001** (35.08)	**< 0.001** (45.02)	**< 0.001** (51.66)

*Normally distributed variables. The *p* value indicates the statistical significance at T2, T3, T4 and T5 compared with T1. GDI, Gait Deviation Index; 6MWT, 6-min walk test; MCID, Minimal Clinically Important Difference; SD, standard deviation; IQR, interquartile range. % ≥ MCID: values represent the percentage of participants whose individual improvement was equal to or greater than the relevant MCID threshold. Statistically significant *p* values are shown in bold.

**Figure 1:**
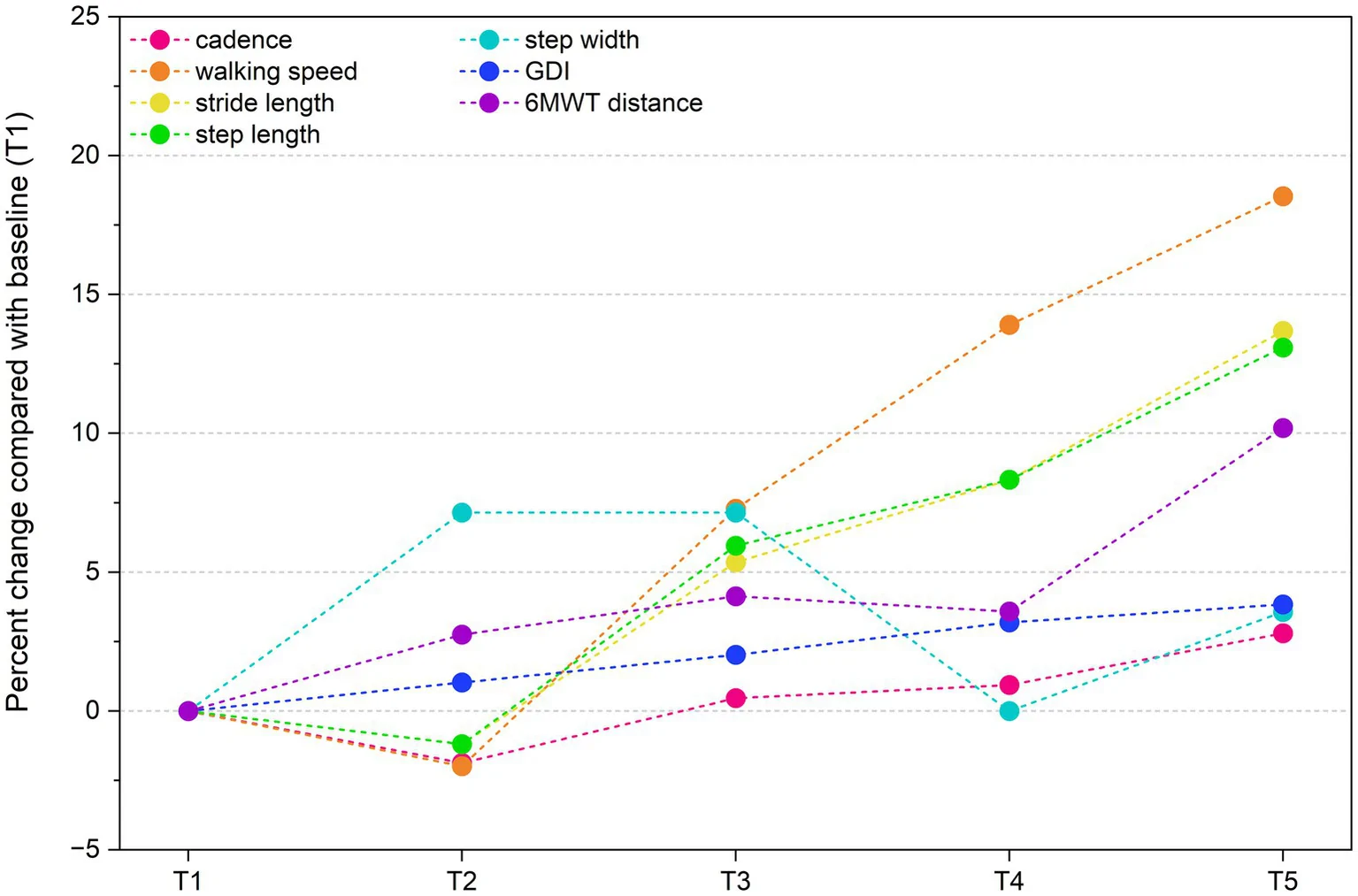
Percentage change from baseline (T1) in the studied parameters for the entire study group.

In the GMFCS I group, walking speed, step length, stride length, and 6MWT distance increased significantly during follow-up (Table 2; Figure 2). Changes in step length, stride length, and 6MWT distance became apparent earlier than changes in walking speed. At T5, individual improvement exceeding the relevant MCID thresholds was observed in 63.64% of participants for walking speed, 76.36% for step length, 74.55% for stride length, and 69.09% for 6MWT distance. GDI increased significantly, although 45.45% of participants exceeded the adopted MCID threshold at T5. No statistically significant changes were observed in cadence or step width.

**Table 2:** Spatiotemporal parameters in the GMFCS I group.

Parameter	MCID	T1	T2	T3	T4	T5	T2 to T1	T3 to T1	T4 to T1	T5 to T1
		Mean (SD) or median (IQR)	Mean (SD) or median (IQR)	Mean (SD) or median (IQR)	Mean (SD) or median (IQR)	Mean (SD) or median (IQR)	*p* value (% ≥ MCID)	*p* value (% ≥ MCID)	*p* value (% ≥ MCID)	*p* value (% ≥ MCID)
Cadence [step/min]	7.15	111.43 (14.30)*	109.64 (16.21)*	111.19 (14.47)*	112.11 (13.16)*	113.13 (14.28)*	0.615 (18.18)	0.894 (27.27)	0.872 (23.64)	0.615 (27.27)
Walking speed [m/s]	0.09	0.88 (0.18)*	0.89 (0.21)*	0.93 (0.19)*	0.98 (0.17)*	1.02 (0.20)*	0.872 (34.55)	0.095 (40.00)	**0.001** (54.55)	**< 0.001** (63.64)
Stride length [m]	0.08	0.95 (0.15)*	0.96 (0.19)*	1.01 (0.16)*	1.05 (0.15)*	1.09 (0.16)*	0.572 (27.27)	**0.005** (41.81)	**< 0.001** (56.36)	**< 0.001** (74.55)
Step length [m]	0.04	0.48 (0.08)*	0.49 (0.09)*	0.50 (0.08)*	0.52 (0.08)*	0.54 (0.08)*	0.217 (34.55)	**0.007** (38.18)	**< 0.001** (58.18)	**< 0.001** (76.36)
Step width [m]	0.02	0.14 (0.05)*	0.15 (0.06)	0.14 (0.06)	0.13 (0.06)	0.14 (0.04)*	0.345 (10.91)	0.393 (21.82)	0.880 (34.55)	0.875 (29.09)
GDI [−]	5.00	85.82 (8.55)*	88.10 (7.66)*	89.34 (12.48)	91.32 (11.66)	92.10 (12.36)	**0.026** (34.55)	**0.009** (36.36)	**0.005** (38.19)	**0.001** (45.45)
6MWT distance [m]	28.04	419.29 (56.09)*	440.56 (67.91)*	445.07 (79.07)*	470.78 (91.10)*	494.60 (102.83)*	**0.004** (49.09)	**0.007** (58.18)	**< 0.001** (69.09)	**< 0.001** (69.09)

^*^Normally distributed variables. The *p* value indicates the statistical significance at T2, T3, T4 and T5 compared with T1. GDI, Gait Deviation Index; 6MWT, 6-min walk test; CID, Minimal Clinically Important Difference; GMFCS, Gross Motor Function Classification System; SD, standard deviation; IQR, interquartile range. % ≥ MCID: values represent the percentage of participants whose individual improvement was equal to or greater than the relevant MCID threshold. Statistically significant *p* values are shown in bold.

**Figure 2:**
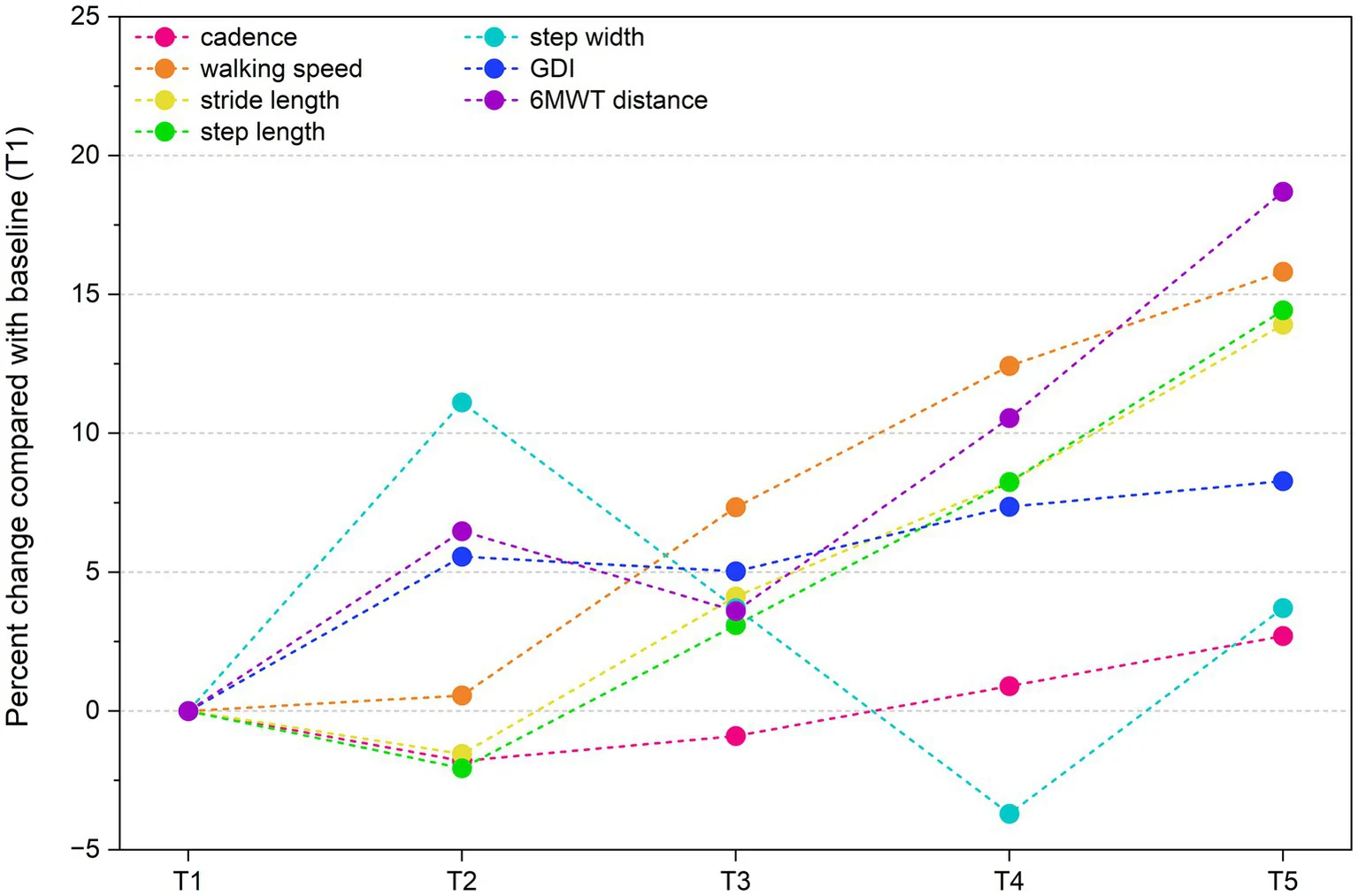
Percentage change from baseline (T1) in the studied parameters for GMFCS I group.

In the GMFCS II group, walking speed, step length, stride length, and 6MWT distance increased significantly during follow-up, although the changes generally became more apparent at the later assessment points (Table 3; Figure 3). At T5, individual improvement exceeding the relevant MCID thresholds was observed in 50.45% of participants for walking speed, 61.07% for step length, 59.30% for stride length, and 53.98% for 6MWT distance. GDI increased significantly at T4 and T5, while 47.79% of participants exceeded the adopted MCID threshold at the final assessment. No statistically significant changes were observed in cadence or step width.

**Table 3:** Spatiotemporal parameters in the GMFCS II group.

Parameter	MCID	T1	T2	T3	T4	T5	T2 to T1	T3 to T1	T4 to T1	T5 to T1
		Mean (SD) or median (IQR)	Mean (SD) or median (IQR)	Mean (SD) or median (IQR)	Mean (SD) or median (IQR)	Mean (SD) or median (IQR)	*p* value (% ≥ MCID)	*p* value (% ≥ MCID)	*p* value (% ≥ MCID)	*p* value (% ≥ MCID)
Cadence [step/min]	10.81	111.02 (21.63)*	109.88 (17.86)*	109.09 (20.94)*	110.00 (22.65)	113.50 (19.50)	0.677 (20.35)	0.677 (18.58)	0.677 (26.55)	0.677 (25.66)
Walking speed [m/s]	0.11	0.77 (0.22)*	0.76 (0.21)*	0.79 (0.24)*	0.88 (0.30)	0.94 (0.32)	0.938 (24.78)	0.337 (32.74)	**0.001** (47.79)	**< 0.001** (50.45)
Stride length [m]	0.08	0.82 (0.17)*	0.83 (0.18)*	0.88 (0.22)	0.91 (0.20)*	0.95 (0.24)	0.389 (27.43)	**0.001** (41.59)	**< 0.001** (57.52)	**< 0.001** (59.30)
Step length [m]	0.04	0.42 (0.11)	0.42 (0.09)*	0.44 (0.11)	0.46 (0.13)	0.48 (0.12)	0.356 (24.78)	**0.001** (38.94)	**< 0.001** (55.75)	**< 0.001** (61.07)
Step width [m]	0.04	0.14 (0.09)	0.16 (0.08)	0.15 (0.08)	0.15 (0.08)	0.15 (0.08)	0.398 (17.70)	0.398 (16.82)	0.713 (16.82)	0.713 (17.70)
GDI [−]	5.00	79.67 (10.14)*	81.03 (10.19)*	81.03 (10.08)*	81.82 (10.09)*	84.09 (13.67)	0.053 (33.63)	0.053 (34.51)	**0.008** (33.63)	**< 0.001** (47.79)
6MWT distance [m]	35.21	365.00 (95.00)	373.38 (81.63)*	378.00 (113.00)	372.00 (95.00)	400.00 (112.00)	**0.003** (39.82)	**0.004** (37.17)	**0.001** (42.48)	**< 0.001** (53.98)

^*^Normally distributed variables. The *p* value indicates the statistical significance at T2, T3, T4 and T5 compared with T1. GDI, Gait Deviation Index; 6MWT, 6-min walk test.

MCID, Minimal Clinically Important Difference; GMFCS, Gross Motor Function Classification System; SD, standard deviation; IQR, interquartile range. % ≥ MCID: values represent the percentage of participants whose individual improvement was equal to or greater than the relevant MCID threshold. Statistically significant *p* values are shown in bold.

**Figure 3:**
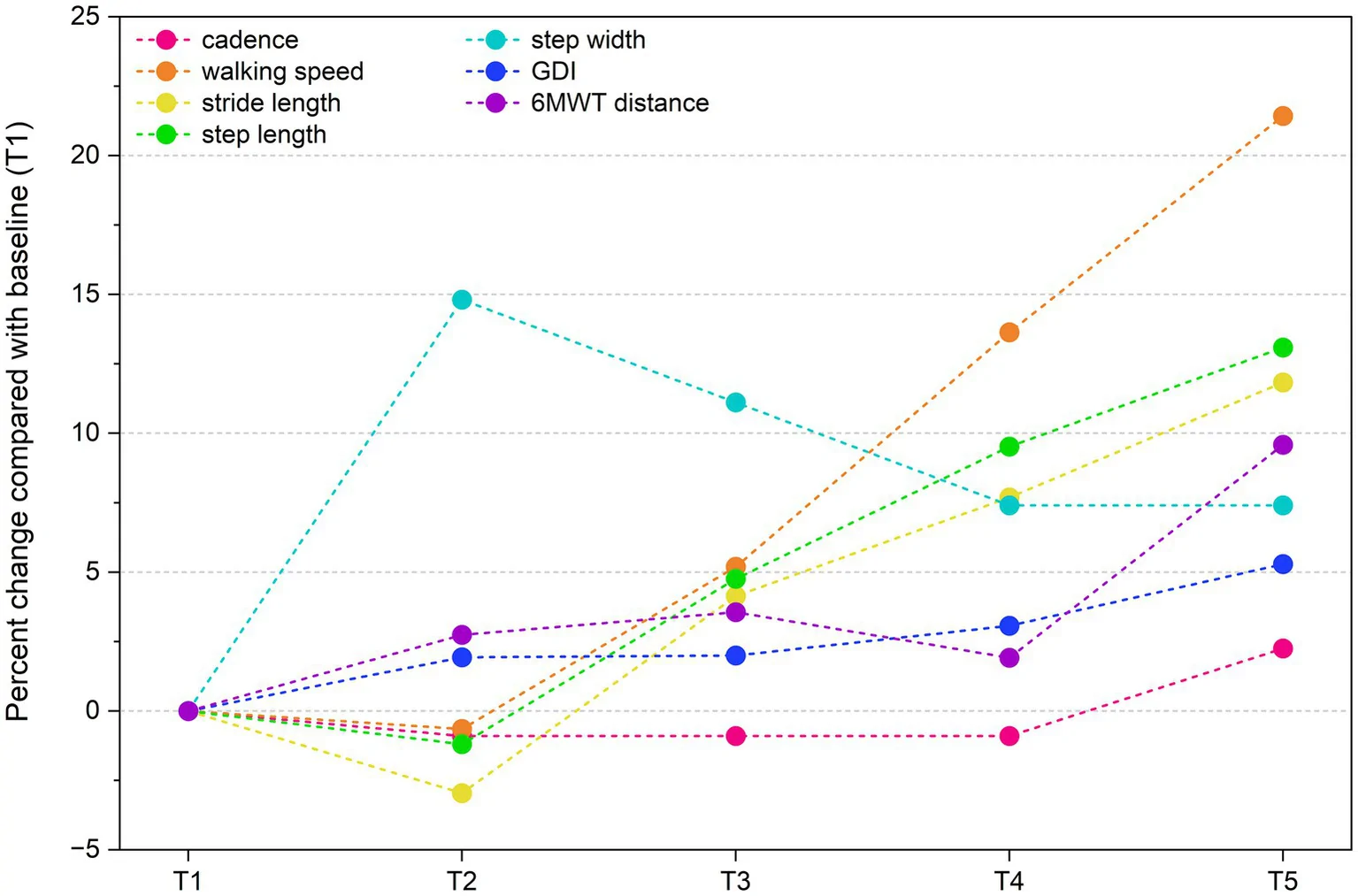
Percentage change from baseline (T1) in the studied parameters for GMFCS II group.

In the GMFCS III group, walking speed, step length, and stride length increased significantly from T2 onward, while 6MWT distance increased significantly at each follow-up assessment (Table 4; Figure 4). At T5, individual improvement exceeding the relevant MCID thresholds was observed in 67.44% of participants for walking speed, 62.79% for step length, 65.12% for stride length, and 60.47% for 6MWT distance. Cadence increased significantly from T3 onward, whereas GDI increased significantly at T4 and T5. However, the highest proportions of participants exceeding the corresponding MCID thresholds were 48.84% for cadence and 46.51% for GDI. Step width remained unchanged.

**Table 4:** Spatiotemporal parameters in the GMFCS III group.

Parameter	MCID	T1	T2	T3	T4	T5	T2 to T1	T3 to T1	T4 to T1	T5 to T1
		Mean (SD) or median (IQR)	Mean (SD) or median (IQR)	Mean (SD) or median (IQR)	Mean (SD) or median (IQR)	Mean (SD) or median (IQR)	*p* value (% ≥ MCID)	*p* value (% ≥ MCID)	*p* value (% ≥ MCID)	*p* value (% ≥ MCID)
Cadence [step/min]	12.75	69.00 (25.49)*	74.73 (24.68)*	77.92 (24.39)*	79.43 (26.27)*	81.38 (26.21)*	0.084 (32.56)	**0.008** (44.19)	**0.008** (39.53)	**0.002** (48.84)
Walking speed [m/s]	0.09	0.34 (0.18)*	0.41 (0.18)*	0.48 (0.22)*	0.50 (0.24)*	0.54 (0.39)	**0.015** (44.19)	**< 0.001** (60.47)	**< 0.001** (51.16)	**< 0.001** (67.44)
Stride length [m]	0.10	0.58 (0.20)*	0.65 (0.20)*	0.72 (0.19)*	0.73 (0.19)*	0.78 (0.19)*	**0.009** (39.53)	**< 0.001** (58.14)	**< 0.001** (51.16)	**< 0.001** (65.12)
Step length [m]	0.05	0.29 (0.10)*	0.33 (0.10)*	0.36 (0.09)*	0.36 (0.10)*	0.39 (0.10)*	**0.006** (48.84)	**< 0.001** (58.14)	**< 0.001** (53.49)	**< 0.001** (62.79)
Step width [m]	0.04	0.16 (0.10)	0.16 (0.06)*	0.16 (0.07)*	0.16 (0.06)*	0.15 (0.05)*	0.891 (18.60)	0.891 (20.93)	0.891 (27.91)	0.891 (20.93)
GDI [−]	5.00	67.16 (8.29)*	67.83 (8.58)*	68.73 (8.16)*	70.12 (8.58)*	71.06 (8.74)*	0.427 (18.61)	0.201 (27.91)	**0.012** (34.88)	**0.025** (46.51)
6MWT distance [m]	44.50	179.44 (89.00)*	225.05 (95.54)*	234.42 (111.65)*	246.16 (96.02)*	246.40 (92.19)*	**< 0.001** (37.21)	**0.001** (48.84)	**< 0.001** (60.47)	**< 0.001** (60.47)

^*^Normally distributed variables. The *p* value indicates the statistical significance at T2, T3, T4 and T5 compared with T1. GDI, Gait Deviation Index; 6MWT, 6-min walk test.

MCID, Minimal Clinically Important Difference; GMFCS, Gross Motor Function Classification System; SD, standard deviation; IQR, interquartile range. % ≥ MCID: values represent the percentage of participants whose individual improvement was equal to or greater than the relevant MCID threshold. Statistically significant *p* values are shown in bold.

**Figure 4:**
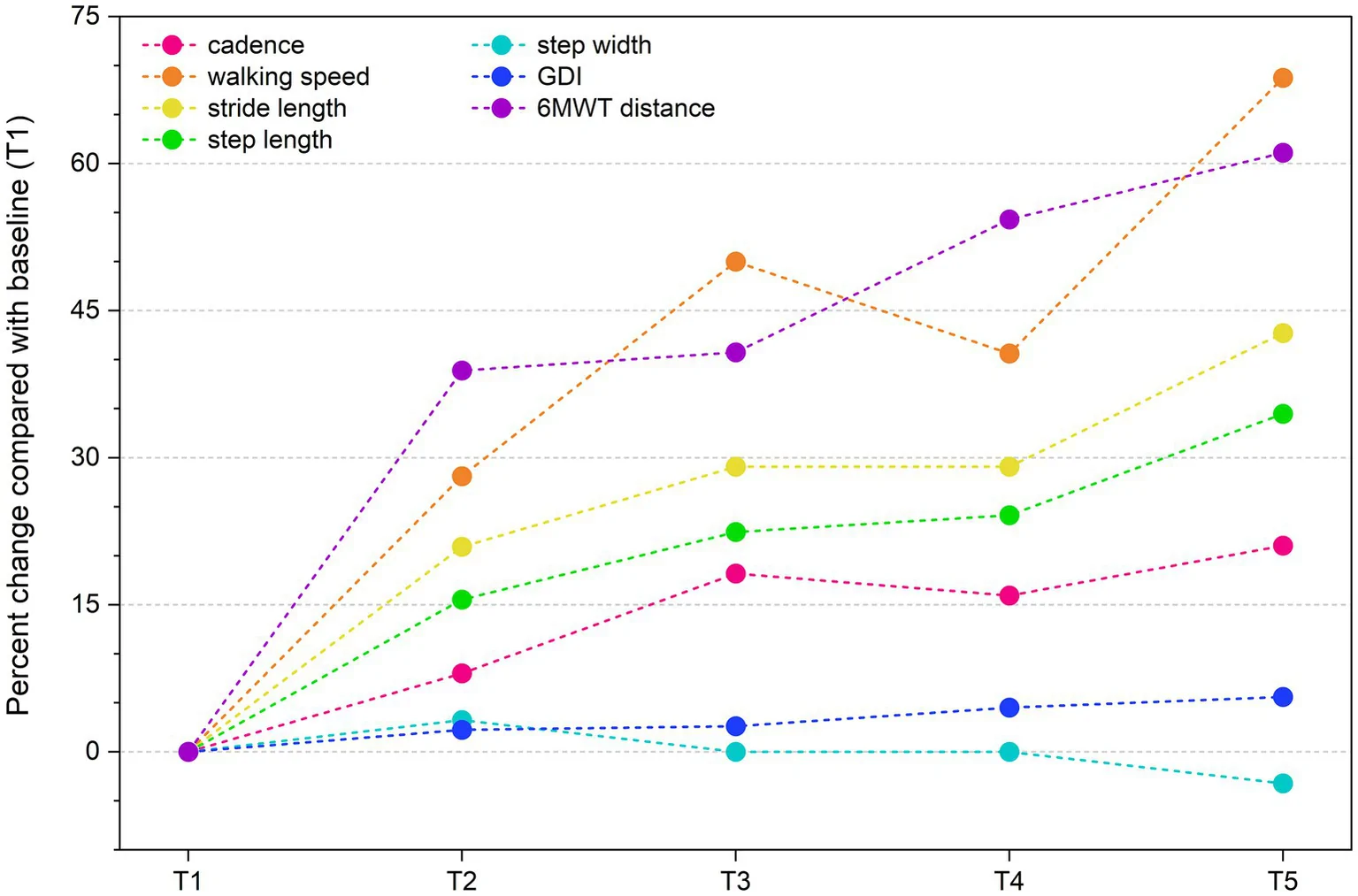
Percentage change from baseline (T1) in the studied parameters for GMFCS III group.

The Supplementary materials provide detailed tables presenting the numerical and percentage classification of changes relative to the MCID, together with figures illustrating longitudinal changes in the studied parameters and the distribution of changes between T5 and T1 with reference to the MCID threshold.

The effect-size analysis showed progressively larger changes relative to baseline for selected gait parameters across the entire study group (Figure 5). Step length and stride length increased significantly from T2 onward, although their effect sizes at T2 were trivial. From T3 onward, walking speed, step length, and stride length showed statistically significant changes with at least small effect sizes, reaching moderate-to-large effect sizes at T5 (Hedges’ *g_z_* = 0.66–0.87). GDI and 6MWT distance increased significantly from T2 onward reaching moderate effect sizes at T5 (Hedges’ *g_z_* = 0.52 and 0.70, respectively). Cadence showed a statistically significant but trivial change only at T5, whereas step width remained unchanged throughout follow-up. Analysis by GMFCS level showed that participants at GMFCS level III demonstrated the earliest and largest gains, including the only significant cadence improvement of any subgroup (from T3). GMFCS I showed a similar but slightly delayed pattern. GMFCS II showed the most modest, later-emerging improvements. Step width stayed unchanged across all subgroups.

**Figure 5:**
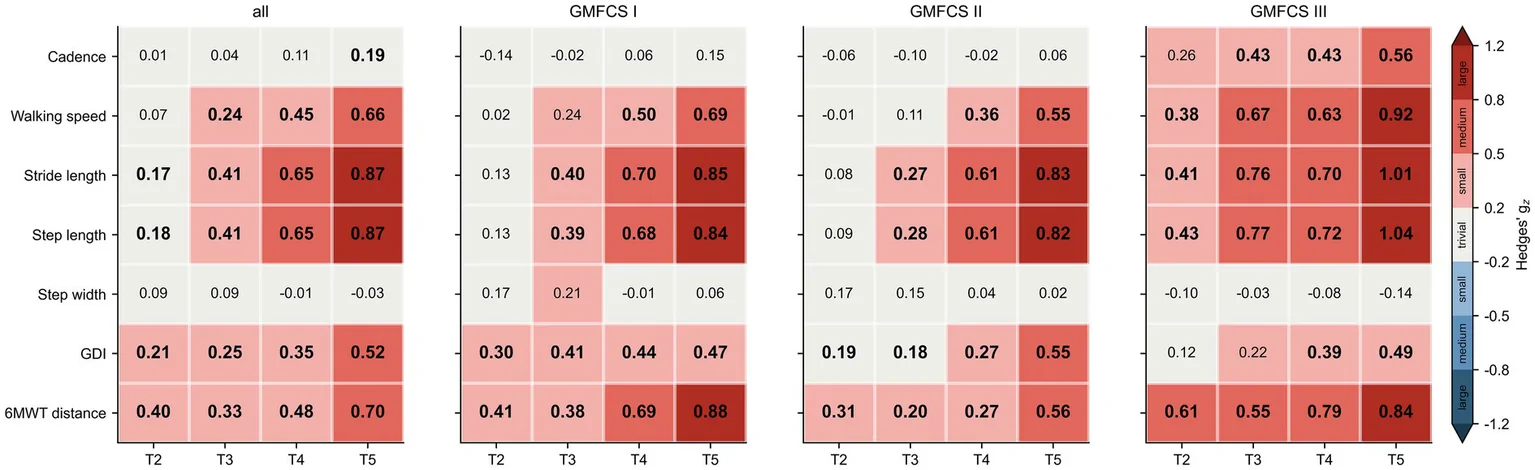
Heatmap of Hedges’ g_z_ effect sizes. Effect sizes at T2, T3, T4, and T5 represent changes compared to baseline (T1). All, entire study group; GMFCS, Gross Motor Function Classification System; GDI, Gait Deviation Index; 6MWT, 6-min walk test. Statistically significant effects are shown in bold and a larger font.

Repeated-measures ANOVA confirmed significant time effects for walking speed, stride length, step length, GDI, and 6MWT distance, strongest in GMFCS I and III (Figure 6A). The time effect on cadence was significant only in the GMFCS III group, and step width showed no significant time effect in any subgroup.

**Figure 6:**
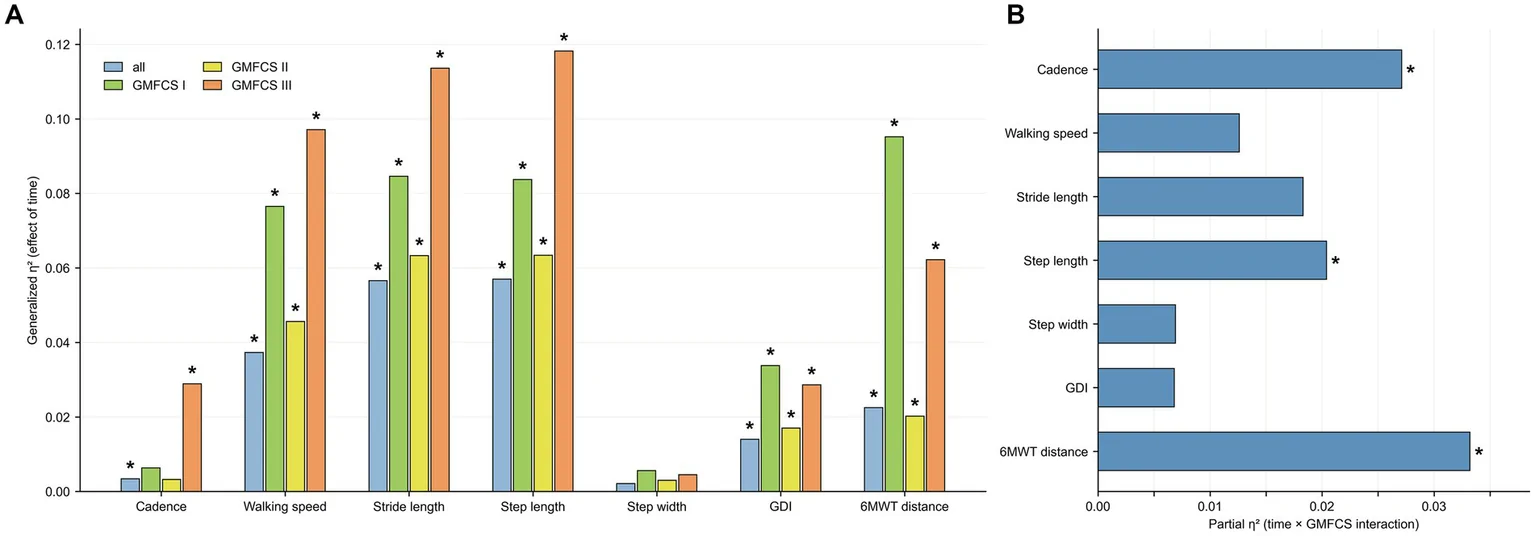
Repeated-measures analysis of variance (ANOVA) results. **(A)** Effect of time on the studied parameters in the entire study group and GMFCS subgroups. **(B)** Time-by-GMFCS interaction for each studied parameter. GMFCS, Gross Motor Function Classification System; GDI, Gait Deviation Index; 6MWT, 6-min walk test. Statistically significant effects are marked with *.

Significant time-by-GMFCS interactions were found for cadence, step length, and 6MWT distance, indicating that the longitudinal trajectories of these parameters differed across GMFCS levels, although the interaction effect sizes were small (Figure 6B). No statistically significant differences in longitudinal trajectories across GMFCS levels were detected for walking speed, stride length, step width, or GDI.

## 4 Discussion

This study aimed to describe changes in spatiotemporal gait parameters and endurance in patients with CP undergoing RAGT, evaluate the dynamics of these changes over time, and determine whether ambulatory level affects responsiveness to RAGT.

We found that patients with CP undergoing repeated RAGT walked faster, took longer steps, and covered greater distances. Statistically significant changes in most of the tested parameters and gait endurance were observed from first follow-up assessment and lasting for 2 years of observation. The responder analysis indicated that improvements in walking speed, step length, stride length, and walking endurance were not limited to changes in group-level values but were also observed at the individual level in a substantial proportion of participants. The timing of these changes varied across ambulatory levels, with selected improvements appearing earlier in participants at GMFCS levels I and III, whereas changes at GMFCS level II generally became more apparent at later assessment points. Importantly, the effect-size analysis showed that the magnitude of change increased over time and was greatest at the final assessment for all statistically significant outcomes. This finding suggests a gradual accumulation of change during the repeated RAGT-based program rather than a uniform response following a single training set. The larger effects observed for selected spatiotemporal parameters at GMFCS level III suggest that responsiveness may differ according to ambulatory level; however, the factors underlying these differences were not directly assessed. In contrast, the consistently negligible effects for step width suggest that this parameter remained stable despite improvements in other aspects of walking performance.

Results from neuroimaging studies suggest that robotic therapy produces modifications in brain activity in both the motor and frontal cortex with an improvement in motor control and attention during RAGT. RAGT may facilitate neural network reorganization through precisely controlled, repetitive movement patterns and may induce adaptive changes in the prefrontal cortex, thereby improving attentional focus and movement trajectory control in children with CP (Perpetuini et al., 2022). One possible explanation for the positive changes observed in the present study is the task-specific nature of RAGT. Growing evidence indicates that repetitive, goal-directed practice can promote neuroplastic changes (Richards et al., 2008). Reorganization of corticospinal motor networks appears to require task-oriented activity rather than isolated training of specific muscles (Kami et al., 1995; Plautz et al., 2000; Rossi et al., 2007). The effectiveness of task-specific training has been demonstrated in individuals with CP (Swinnen et al., 2010; Beretta et al., 2020), as well as in individuals with traumatic brain injury (Chua et al., 2007) and stroke (Sullivan et al., 2007). Although positive changes in brain activity have been demonstrated, their direct impact on gait function remains unclear. Since the present study did not include neurophysiological measurements, these mechanisms should be regarded as possible explanations rather than confirmed mechanisms underlying the observed changes. Analysis of existing studies investigating the impact of RAGT on gait has shown inconclusive results. While some studies have shown significant short- and long-term improvements in spatiotemporal parameters and endurance (Borggraefe et al., 2010; Smania et al., 2011; Yazıcı et al., 2019; Drużbicki et al., 2013; Sarhan et al., 2014; Wu et al., 2017; Meyer-Heim et al., 2007, 2009), other studies reported no significant changes in the assessed gait outcomes (Chen et al., 2026; Ammann-Reiffer et al., 2020; Jin et al., 2020; Patritti et al., 2010; Schroeder et al., 2014; van Hedel et al., 2016). A more recent systematic review found that RAGT led to clinically meaningful improvements in gait speed, other gait parameters and functionality in individuals with CP, with effects sustained at a long-term follow-up (Volpini et al., 2022). Moreover, most studies based on individual trials reported some positive benefits of RAGT in children with CP on activity parameters such as standing ability, walking speed, and distance; however, a meta-analysis did not confirm this finding (Lefmann et al., 2017).

Our study focused only on sustained, clinically meaningful changes in gait and endurance achieved through repeated RAGT sets. While majority of previous studies primarily reported results from a single RAGT set with follow-up limited to 6 months, the final assessment in the present study was performed approximately 6 months after the fourth RAGT set, with a total follow-up of 2 years from treatment initiation.

Given the mean baseline age of the participants, functional trajectories in CP were expected to have largely stabilized. Although natural growth and motor maturation may have contributed to the observed changes, baseline values were close to previously reported functional limits, whereas the final values of selected outcomes exceeded the expected plateau values. In addition, participants continued the same conventional therapy they had received before enrolment. Taken together, these observations suggest that the longitudinal changes may extend beyond the expected developmental course and are consistent with an additional contribution of RAGT.

For children with no history of surgery, the developmental trajectory of the 6MWT showed the greatest gains between 3 and 5 years of age, with an estimated functional limit reached by age 12. The functional limits differed according to GMFCS level and were consistent with gross motor function limits, which were 417 m for participants at level I, 342 m at level II, and 180.7 m at level III. Moreover, the age at which 90% of the estimated final value was achieved differed depending on ambulatory skills. On average, this was over 50 months for level I and 69 months for level II – taking longer to develop strength and balance than level I – while level III achieved functional growth earliest, at 20 months. These findings suggest that intervention focused on walking capacity should begin as early as age 3 to facilitate functional mobility (Fiss et al., 2019).

In our study, the mean age of participants at first assessment was 10.33 years, and patients at each ambulatory level presented estimated functional limits (level I: 419.3 m; level II: 365 m, level III: 179.4 m). After 2 years of follow-up, 6MWT distance had increased by 75.3 m at GMFCS level I, 35 m at level II, and 67 m at level III. The significant time-by-GMFCS interaction indicates that the longitudinal trajectory of 6MWT distance differed across functional levels. A substantial proportion of participants at GMFCS levels I and III showed individual improvement exceeding the study-specific MCID threshold already after 12 months, whereas at GMFCS level II this proportion increased mainly during the later stages of follow-up, reaching the highest value at the final assessment.

The largest relative increase in 6MWT distance (37%) was observed in patients at GMFCS level III. Considering that participants entered the study at an age when functional trajectories in CP, particularly at lower functional levels, are expected to have stabilized, the observed improvement may extend beyond the expected developmental course (Beckung et al., 2007; Fiss et al., 2019). However, it should not be interpreted as evidence that RAGT was more effective at GMFCS level III. As previously suggested (Fiss et al., 2019), individuals at GMFCS level II may require more time to develop the strength and balance necessary for improved walking endurance, which may partly explain the later changes observed in this group. The significant time-by-GMFCS interactions for 6MWT distance, cadence, and step length further indicate that the trajectories of selected outcomes differed across functional levels. However, the small interaction effect sizes suggest that these differences were limited and did not represent a general pattern across all gait outcomes.

We have also found improvements in spatiotemporal parameters; however, only for walking speed, step length, and stride length did the majority of participants exceed the relevant MCID thresholds, with these parameters showing moderate-to-large effect sizes. It has been shown that with age, patients with CP walk at a slower speed (based on the average change between baseline and final assessment), while step and stride length remain unchanged (Daly et al., 2022). In our study, all participants walked faster with longer step and stride length, which appear to demonstrate beneficial effects of RAGT and deviate from the natural age-related progression described above (Daly et al., 2022).

We observed small changes in GDI that reached statistical significance; however, at the final assessment, individual improvement exceeding the adopted MCID threshold was observed in approximately 47% of participants, with similar proportions across the GMFCS subgroups (45.45% at GMFCS level I, 47.79% at level II, and 46.51% at level III). This apparent discrepancy may reflect the specific characteristics of motor adaptation in individuals with CP. A few studies have examined whether changes in GDI correspond to changes in walking performance or endurance, and the available evidence remains inconclusive (Bonnefoy-Mazure et al., 2020; Joanna et al., 2020; White et al., 2023). Improvements in spatiotemporal gait parameters may occur more frequently than improvements in GDI, although concurrent changes in GDI and selected gait parameters have also been reported. Functional improvements may occur without a corresponding change exceeding the GDI MCID threshold, as increased walking speed or endurance may result from changes in strength, balance, walking tolerance, or compensatory strategies that do not normalize the global kinematic pattern captured by GDI. Moreover, as a composite measure, GDI may not be sufficiently sensitive to detect subtle or localized kinematic adaptations that contribute to improved walking performance. Therefore, improvement exceeding the GDI MCID threshold may not be a prerequisite for improvements in walking speed or endurance in individuals with CP. This interpretation is consistent with previous investigations of the natural, age-related progression of GDI, which reported marginal changes that remained below the threshold for a clinically meaningful difference (Chen et al., 2026; Daly et al., 2022; Rose et al., 2010). The additional analyses confirmed a statistically significant effect of time on GDI within each GMFCS level, while the absence of a significant time-by-GMFCS interaction indicated that its longitudinal trajectory did not differ significantly across functional levels. Thus, GDI showed a consistent but limited change across the study population.

We propose that the critical knowledge gap concerns the optimal RAGT dose, including the number of sessions and treatment duration required to achieve sustained changes in gait parameters and walking endurance. Further research should also determine when clinically meaningful changes can be expected and which patient profiles are most likely to benefit.

Existing RAGT studies predominantly focus on low-dose interventions leaving the dose–response relationship for moderate- and long-term RAGT underexplored. A recent meta-analysis showed that medium-term interventions (8 weeks) yield superior outcomes (Chen et al., 2026). In the present study, changes after the initial RAGT set were generally small, whereas larger and more consistent changes became evident after subsequent sets and during later assessment. Previous studies focused on short- or long-term changes, but observation periods did not exceed 6 months after a single set of intervention. The effect-size analysis showed that the magnitude of change increased over time and reached its largest value at T5 for all statistically significant outcomes. This pattern suggests that changes accumulated progressively during the repeated RAGT-based program.

Additionally, our study aimed to determine whether the timing and magnitude of change differed across ambulatory levels. Previous studies have reported the use of RAGT in patients classified at GMFCS levels I-IV; however, differences in treatment outcomes by ambulatory level have not been investigated. Moreover, it is well known that children with CP achieve their expected functional level well before adolescence. The plateau is observed earliest in children walking with aids, at the lower functional level (Beckung et al., 2007; Fiss et al., 2019). Changes were observed across all GMFCS levels, although their timing and magnitude varied for selected outcomes. Selected spatiotemporal changes emerged relatively early in participants at GMFCS level III, whereas the proportion of responders at GMFCS level II generally increased at the later assessments, particularly at T4 and T5. Our study suggests that improvements in gait and endurance may occur in patients at all ambulatory levels, even though they have already achieved their expected endurance and functional skills, and given their age, are expected to have reached a plateau.

Our findings suggest that repeated, longitudinal RAGT may be associated with progressive and sustained improvements in selected gait parameters and walking endurance. The relatively small changes observed after the first RAGT set, followed by larger changes after subsequent blocks, indicate that a longer treatment and observation period may be required to detect clinically meaningful improvement.

### 4.1 Limitations

Several methodological aspects should be considered when interpreting these findings, relating to the assessment of clinical relevance, the variability of RAGT equipment, and the observational study design.

One limitation concerns the assessment of clinical relevance: no external anchors of perceived or functional change, participation, or quality of life were available. Nevertheless, the distribution-based MCID thresholds provided a consistent framework for classifying individual changes relative to study-specific thresholds across the analyzed parameters, rather than direct measures of patient-perceived benefit. Future studies incorporating external anchors could determine more fully whether the observed gait-related changes translate into improvements in everyday functioning and participation.

Another limitation of the study relates to the variability of RAGT equipment used. Different RAGT devices were required because of variation in participants’ body dimensions, functional abilities, and therapeutic needs. This is consistent with previous analyses of RAGT effectiveness, which have similarly pooled data across multiple equipment types and therapeutic protocols (Lefmann et al., 2017; Cortés-Pérez et al., 2022; Volpini et al., 2022; Chen et al., 2026). In our study, by contrast, device selection was based on compatibility with the technical specifications of each system and individualized clinical requirements, whereas the structure, duration, and frequency of the therapeutic program remained standardized. Complete longitudinal data on individual device exposure and device-specific settings were not available, so the influence of device type could not be formally assessed. Accordingly, the findings relate to the repeated RAGT-based program as a whole rather than to the effectiveness of any specific device.

A further limitation was the lack of randomization and a concurrent control group. However, several factors support the interpretation that the observed improvements may extend beyond expected developmental change alone. First, participants generally entered the study at an age when functional trajectories in CP tend to stabilize, particularly at lower functional levels (Beckung et al., 2007; Fiss et al., 2019). Second, the observed changes were interpreted in relation to previously published age- and GMFCS-specific developmental trajectories for walking endurance and spatiotemporal gait parameters, and the final values exceeded the expected plateau values for selected outcomes (Fiss et al., 2019; Daly et al., 2022). Third, participants were already receiving conventional therapy at baseline, and this therapy continued without interruption or modification throughout the follow-up period, reducing the likelihood that changes in conventional care accounted for the observed longitudinal improvements. Taken together, these observations suggest that the observed improvements are unlikely to be fully explained by maturation or ongoing conventional therapy alone and are consistent with an additional contribution of RAGT.

## 5 Conclusion

Systematic, repetitive gait training based on RAGT in patients with CP was associated with long-term positive changes in spatiotemporal gait parameters and walking endurance. In contrast, clinically meaningful changes in gait kinematics, as quantified by the GDI, were limited across all GMFCS levels. The effects of systematic and repeated RAGT developed progressively over time, with selected outcomes following different trajectories according to the participants’ functional level.

## Data Availability

The raw data supporting the conclusions of this article will be made available by the authors, without undue reservation.
