# Supplementary material for: Long-term robot-assisted gait training in patients with cerebral palsy: meaningful changes in gait parameters require time
Source: Front Hum Neurosci. 2026 Sep 24;20:1924639. doi: 10.3389/fnhum.2026.1924639 (PMC13646091; doi:10.3389/fnhum.2026.1924639)
Supplement: Supplementary file 1 [file Data_Sheet_1.DOCX]

Supplementary Material

**Table S1.** MCID-based changes in the entire group over time

| Parameter | MCID | T2 to T1 N (% of group) | | | T3 to T1 N (% of group) | | | T4 to T1 N (% of group) | | | T5 to T1 N (% of group) | | |
| --- | --- | --- | --- | --- | --- | --- | --- | --- | --- | --- | --- | --- | --- |
|  |  | Up | No change | Down | Up | No change | Down | Up | No change | Down | Up | No change | Down |
| Cadence [step/min] | 10.29 | 45 (21.33) | 115 (54.50) | 51 (24.17) | 53 (25.12) | 111 (52.61) | 47 (22.27) | 61 (28.91) | 98 (46.45) | 52 (24.64) | 68 (32.23) | 102 (48.34) | 41 (19.43) |
| Walking speed [m/s] | 0.14 | 55 (26.07) | 115 (54.50) | 41 (19.43) | 63 (29.86) | 114 (54.03) | 34 (16.11) | 90 (42.65) | 95 (45.02) | 26 (12.33) | 110 (52.13) | 83 (39.34) | 18 (8.53) |
| Stride length [m] | 0.10 | 56 (26.54) | 115 (54.50) | 40 (18.96) | 84 (39.81) | 103 (48.82) | 24 (11.37) | 108 (51.18) | 82 (38.87) | 21 (9.95) | 131 (62.09) | 66 (31.27) | 14 (6.64) |
| Step length [m] | 0.05 | 63 (29.86) | 111 (52.60) | 37 (17.54) | 86 (40.76) | 100 (47.39) | 25 (11.85) | 112 (53.08) | 75 (35.55) | 24 (11.37) | 130 (61.61) | 69 (32.70) | 12 (5.69) |
| Step width [m] | 0.03 | 36 (17.06) | 120 (56.87) | 55 (26.07) | 41 (19.43) | 112 (53.08) | 58 (27.49) | 48 (22.75) | 113 (53.55) | 50 (23.70) | 41 (19.43) | 129 (61.14) | 41 (19.43) |
| GDI [-] | 5.00 | 65 (30.80) | 111 (52.61) | 35 (16.59) | 71 (33.65) | 105 (49.76) | 35 (16.59) | 74 (35.07) | 103 (48.82) | 34 (16.11) | 99 (46.92) | 89 (42.18) | 23 (10.90) |
| 6MWT distance [m] | 51.70 | 64 (30.33) | 126 (59.72) | 21 (9.95) | 74 (35.08) | 113 (53.55) | 24 (11.37) | 95 (45.02) | 97 (45.97) | 19 (9.01) | 109 (51.66) | 88 (41.70) | 14 (6.64) |

**: normally distributed variables. GDI: Gait Deviation Index; 6MWT: 6-minute walk test.
MCID: Minimal Clinically Important Difference; Categories are based on MCID thresholds (Up* ≥ *MCID; Down* ≤ −*MCID; No change: |*Δ| < *MCID).
Values indicates the clinical changes at T2, T3, T4 and T5 compared with T1.*

**Table S2.** MCID-based changes in GMFCS I group over time

| Parameter | MCID | T2 to T1 N (% of group) | | | T3 to T1 N (% of group) | | | T4 to T1 N (% of group) | | | T5 to T1 N (% of group) | | |
| --- | --- | --- | --- | --- | --- | --- | --- | --- | --- | --- | --- | --- | --- |
|  |  | Up | No change | Down | Up | No change | Down | Up | No change | Down | Up | No change | Down |
| Cadence [step/min] | 7.15* | 10 (18.18) | 27 (49.09) | 18 (32.73) | 15 (27.27) | 25 (45.46) | 15 (27.27) | 13 (23.64) | 28 (50.91) | 14 (25.45) | 15 (27.27) | 29 (52.73) | 11 (20.00) |
| Walking speed [m/s] | 0.09* | 19 (34.55) | 20 (36.36) | 16 (29.09) | 22 (40.00) | 23 (41.82) | 10 (18.18) | 30 (54.55) | 17 (30.90) | 8 (14.55) | 35 (63.64) | 16 (29.09) | 4 (7.27) |
| Stride length [m] | 0.08* | 15 (27.27) | 28 (50.91) | 12 (21.82) | 23 (41.81) | 24 (43.64) | 8 (14.55) | 31 (56.36) | 19 (34.55) | 5 (9.09) | 41 (74.55) | 10 (18.18) | 4 (7.27) |
| Step length [m] | 0.04* | 19 (34.55) | 24 (43.64) | 12 (21.81) | 21 (38.18) | 27 (49.09) | 7 (12.73) | 32 (58.18) | 17 (30.91) | 6 (10.91) | 42 (76.36) | 9 (16.37) | 4 (7.27) |
| Step width [m] | 0.02* | 6 (10.91) | 33 (60.00) | 16 (29.09) | 12 (21.82) | 27 (49.09) | 16 (29.09) | 19 (34.55) | 21 (38.18) | 15 (27.27) | 16 (29.09) | 21 (38.18) | 18 (32.73) |
| GDI [-] | 5.00 | 19 (34.55) | 27 (49.09) | 9 (16.36) | 20 (36.36) | 29 (52.73) | 6 (10.91) | 21 (38.19) | 25 (45.45) | 9 (16.36) | 25 (45.45) | 24 (43.64) | 6 (10.91) |
| 6MWT distance [m] | 28.04* | 27 (49.09) | 19 (34.55) | 9 (16.36) | 32 (58.18) | 12 (21.82) | 11 (20.00) | 38 (69.09) | 11 (20.00) | 6 (10.91) | 38 (69.09) | 13 (23.64) | 4 (7.27) |

**: normally distributed variables. GDI: Gait Deviation Index; 6MWT: 6-minute walk test; GMFCS: Gross Motor Function Classification System.
MCID: Minimal Clinically Important Difference; Categories are based on MCID thresholds (Up* ≥ *MCID; Down* ≤ −*MCID; No change: |*Δ| < *MCID).
Values indicates the clinical changes at T2, T3, T4 and T5 compared with T1.*

**Table S3.** MCID-based changes in GMFCS II group over time

| Parameter | MCID | T2 to T1 N (% of group) | | | T3 to T1 N (% of group) | | | T4 to T1 N (% of group) | | | T5 to T1 N (% of group) | | |
| --- | --- | --- | --- | --- | --- | --- | --- | --- | --- | --- | --- | --- | --- |
|  |  | Up | No change | Down | Up | No change | Down | Up | No change | Down | Up | No change | Down |
| Cadence [step/min] | 10.81* | 23 (20.35) | 61 (53.99) | 29 (25.66) | 21 (18.58) | 62 (54.87) | 30 (26.55) | 30 (26.55) | 48 (42.48) | 35 (30.97) | 29 (25.66) | 58 (51.33) | 26 (23.01) |
| Walking speed [m/s] | 0.11* | 28 (24.78) | 51 (45.13) | 34 (30.09) | 37 (32.74) | 47 (41.59) | 29 (26.67) | 54 (47.79) | 36 (31.86) | 23 (20.35) | 57 (50.45) | 43 (38.05) | 13 (11.50) |
| Stride length [m] | 0.08* | 31 (27.43) | 57 (50.45) | 25 (22.12) | 47 (41.59) | 48 (42.48) | 18 (15.93) | 65 (57.52) | 34 (30.09) | 14 (12.39) | 67 (59.30) | 37 (32.74) | 9 (7.96) |
| Step length [m] | 0.04 | 28 (24.78) | 62 (54.87) | 23 (20.35) | 44 (38.94) | 51 (45.13) | 18 (15.93) | 63 (55.75) | 34 (30.09) | 16 (14.16) | 69 (61.07) | 35 (30.97) | 9 (7.96) |
| Step width [m] | 0.04 | 20 (17.70) | 66 (58.41) | 27 (23.89) | 19 (16.82) | 67 (59.29) | 27 (23.89) | 19 (16.82) | 73 (64.60) | 21 (18.58) | 20 (17.70) | 76 (67.26) | 17 (15.04) |
| GDI [-] | 5.00 | 38 (33.63) | 55 (48.67) | 20 (17.70) | 39 (34.51) | 52 (46.02) | 22 (19.47) | 38 (33.63) | 54 (47.79) | 21 (18.58) | 54 (47.79) | 47 (41.59) | 12 (10.62) |
| 6MWT distance [m] | 35.21 | 45 (39.82) | 48 (42.48) | 20 (17.70) | 42 (37.17) | 51 (45.13) | 20 (17.70) | 48 (42.48) | 48 (42.48) | 17 (15.04) | 61 (53.98) | 37 (32.75) | 15 (13.27) |

**: normally distributed variables. GDI: Gait Deviation Index; 6MWT: 6-minute walk test; GMFCS: Gross Motor Function Classification System.
MCID: Minimal Clinically Important Difference; Categories are based on MCID thresholds (Up* ≥ *MCID; Down* ≤ −*MCID; No change: |*Δ| < *MCID).
Values indicates the clinical changes at T2, T3, T4 and T5 compared with T1.*

**Table S4.** MCID-based changes in GMFCS III group over time

| Parameter | MCID | T2 to T1 N (% of group) | | | T3 to T1 N (% of group) | | | T4 to T1 N (% of group) | | | T5 to T1 N (% of group) | | |
| --- | --- | --- | --- | --- | --- | --- | --- | --- | --- | --- | --- | --- | --- |
|  |  | Up | No change | Down | Up | No change | Down | Up | No change | Down | Up | No change | Down |
| Cadence [step/min] | 12.75* | 14 (32.56) | 21 (48.84) | 8 (18.60) | 19 (44.19) | 19 (44.19) | 5 (11.62) | 17 (39.53) | 18 (41.87) | 8 (18.60) | 21 (48.84) | 16 (37.21) | 6 (13.95) |
| Walking speed [m/s] | 0.09* | 19 (44.19) | 18 (41.86) | 6 (13.95) | 26 (60.47) | 13 (30.23) | 4 (9.30) | 22 (51.16) | 15 (34.89) | 6 (13.95) | 29 (67.44) | 11 (25.58) | 3 (6.98) |
| Stride length [m] | 0.10* | 17 (39.53) | 20 (46.52) | 6 (13.95) | 25 (58.14) | 15 (34.88) | 3 (6.98) | 22 (51.16) | 16 (37.21) | 5 (11.62) | 28 (65.12) | 13 (30.23) | 2 (4.65) |
| Step length [m] | 0.05* | 21 (48.84) | 15 (34.88) | 7 (16.28) | 25 (58.14) | 15 (34.88) | 3 (6.98) | 23 (53.49) | 16 (37.21) | 4 (9.30) | 27 (62.79) | 14 (32.56) | 2 (4.65) |
| Step width [m] | 0.04 | 8 (18.60) | 25 (58.14) | 10 (23.26) | 9 (20.93) | 19 (44.19) | 15 (34.88) | 12 (27.91) | 17 (39.53) | 14 (32.56) | 9 (20.93) | 24 (55.81) | 10 (23.26) |
| GDI [-] | 5.00 | 8 (18.61) | 29 (67.44) | 6 (13.95) | 12 (27.91) | 24 (55.81) | 7 (16.28) | 15 (34.88) | 24 (55.81) | 4 (9.31) | 20 (46.51) | 18 (41.86) | 5 (11.63) |
| 6MWT distance [m] | 44.50* | 16 (37.21) | 23 (53.49) | 4 (9.30) | 21 (48.84) | 16 (37.21) | 6 (13.95) | 26 (60.47) | 14 (32.55) | 3 (6.98) | 26 (60.47) | 13 (30.23) | 4 (9.30) |

**: normally distributed variables. GDI: Gait Deviation Index; 6MWT: 6-minute walk test; GMFCS: Gross Motor Function Classification System.
MCID: Minimal Clinically Important Difference; Categories are based on MCID thresholds (Up* ≥ *MCID; Down* ≤ −*MCID; No change: |*Δ| < *MCID).
Values indicates the clinical changes at T2, T3, T4 and T5 compared with T1.*

**Supplementary Figure 1.** Changes in cadence over time. Cadence values at each assessment point in each group (A); distribution of changes in cadence between T5 and T1 in the: entire study group (B), GMFCS I group (C); GMFCS II group (D); GMFCS III group (E).

**A**

**B**

**C**

**D**

**E**

*all: entire study group; IQR: interquartile range; GMFCS: Gross Motor Function Classification System;* Δ*: change from T1 to T5. The shaded areas represent change less than the minimal clinically important difference (MCID). The solid vertical lines represent zero change and the dashed lines represent mean change.*

**Supplementary** **Figure 2.** Changes in walking speed over time. Walking speed values at each assessment point in each group (A); distribution of changes in walking speed between T5 and T1 in the: entire study group (B), GMFCS I group (C), GMFCS II group (D), GMFCS III group (E).

**A**

**B**

**C**

**D**

**E**

*all: entire study group; IQR: interquartile range; GMFCS: Gross Motor Function Classification System;* Δ*: change from T1 to T5. The shaded areas represent change less than the minimal clinically important difference (MCID). The solid vertical lines represent zero change and the dashed lines represent mean change.*

**Supplementary** **Figure 3.** Changes in stride length over time. Stride length values at each assessment point in each group (A); distribution of changes in stride length between T5 and T1 in the: entire study group (B), GMFCS I group (C), GMFCS II group (D), GMFCS III group (E).

**A**

**B**

**C**

**D**

**E**

*all: entire study group; IQR: interquartile range; GMFCS: Gross Motor Function Classification System;* Δ*: change from T1 to T5. The shaded areas represent change less than the minimal clinically important difference (MCID). The solid vertical lines represent zero change and the dashed lines represent mean change.*

**Supplementary** **Figure 4.** Changes in step length over time. Step length values at each assessment point in each group (A); distribution of changes in step length between T5 and T1 in the: entire study group (B), GMFCS I group (C), GMFCS II group (D), GMFCS III group (E).

**A**

**B**

**C**

**D**

**E**

*all: entire study group; IQR: interquartile range; GMFCS: Gross Motor Function Classification System;* Δ*: change from T1 to T5. The shaded areas represent change less than the minimal clinically important difference (MCID). The solid vertical lines represent zero change and the dashed lines represent mean change.*

**Supplementary** **Figure 5.** Changes in step width over time. Step width values at each assessment point in each group (A); distribution of changes in step width between T5 and T1 in the: entire study group (B), GMFCS I group (C), GMFCS II group (D), GMFCS III group (E).

**A**

**B**

**C**

**D**

**E**

*all: entire study group; IQR: interquartile range; GMFCS: Gross Motor Function Classification System;* Δ*: change from T1 to T5. The shaded areas represent change less than the minimal clinically important difference (MCID). The solid vertical lines represent zero change and the dashed lines represent mean change.*

**Supplementary** **Figure 6.** Changes in GDI over time. GDI values at each assessment point in each group (A); distribution of changes in GDI between T5 and T1 in the: entire study group (B), GMFCS I group (C), GMFCS II group (D), GMFCS III group (E).

**A**

**B**

**C**

**D**

**E**

*GDI: Gait Deviation Index; all: entire study group; IQR: interquartile range; GMFCS: Gross Motor Function Classification System;* Δ*: change from T1 to T5. The shaded areas represent change less than the minimal clinically important difference (MCID). The solid vertical lines represent zero change and the dashed lines represent mean change.*

**Supplementary** **Figure 7.** Changes in 6MWT distance over time. 6MWT distance values at each assessment point in each group (A); distribution of changes in 6MWT distance between T5 and T1 in the: entire study group (B), GMFCS I group (C), GMFCS II group (D), GMFCS III group (E).

**A**

**B**

**C**

**D**

**E**

*6MWT: 6-minute walk test; all: entire study group; IQR: interquartile range; GMFCS: Gross Motor Function Classification System;* Δ*: change from T1 to T5. The shaded areas represent change less than the minimal clinically important difference (MCID). The solid vertical lines represent zero change and the dashed lines represent mean change.*
